# ZnO-Salen NPs Employed as Chemosensor for Detection of Al^3+^ and K^+^ in Aqueous Medium, Developing Human Cell Images

**DOI:** 10.1007/s10895-024-03913-4

**Published:** 2024-08-31

**Authors:** Carlos Alberto Huerta-Aguilar, Iván J. Bazany-Rodríguez, Valeria Hansberg-Pastor, Ignacio Camacho-Arroyo, Iván Alejandro Reyes-Dominguez, Pabel Antonio Cervantes-Avilés, Pandiyan Thangarasu

**Affiliations:** 1 School of Engineering and Sciences, Tecnológico de Monterrey, Puebla, 72456 Puebla Mexico; 2 Faculty of Chemistry, Universidad Nacional Autónoma de Mexico, CDMX, Mexico City, 04510 Mexico; 3 Instituto Nacional de Perinatología-Facultad de Química, Unidad de Investigación en Reproducción Humana, Universidad Nacional Autónoma de México, CDMX, Mexico City, 04510 Mexico; 4 Instituto de Metalurgia, Universidad Autónoma de San Luis Potosí, San Luis Potosí, 78210 Mexico

**Keywords:** Chemosensor, ZnO nanoparticles, Fluorescence, Aluminium, Potassium

## Abstract

ZnO nanoparticles (NPs) were prepared and characterized by different analytical methods and then they were used to decorate with *N*,* N*´-bis(salicylidene)ethylenediamine (salen) in order to perform as receptor for the metal ions in an aqueous medium. The results show that ZnO-salen selectively detects Al^3+^ ions in aqueous medium since the intensity of fluorescence has been enhanced significantly. However, the presence of K^+^ in the medium further intensified the fluorescence emission for the [ZnO-salen-Al^3+^] system. The above system has been applied to recognize Al^3+^ and K^+^ in cells by developing the cell images, for which, the fluorescence image is brightened if a human glioblastoma U251 cell contains [ZnO-salen-Al^3+^] + K^+^ ions, consisting of the fluorescence titration. The binding global constant for Al^3+^ and the subsequent recognition of K^+^ by ZnO-salen resulted in *β*_*2*_(Al^3+^) = 6.61 × 10^3^ and *β*_*2*_(K^+^) = 3.71 × 10^3^ with a detection limit of 36.51 µM for Al^3+^ and 17.39 µM for K^+^. In the cell toxicity analysis, the cell viability was over 85% for the ZnO-salen even in the concentration as high as 100 mM.

**Supplementary Information:**

The online version contains supplementary material available at 10.1007/s10895-024-03913-4.

## Introduction

Highly selective fluorescent sensors are interesting in the view of detection and quantification of various metal ions or anions in the biological and environmental samples [1]. So, the development of such molecular devices is attractive since their distinct optical properties are of great interest and the specific interaction of fluorophores (host or receptor) with analytes (guests = cations or anions) can undergo a substantial conformational change in order to enhance or suppress the florescence intensities; i.e. the binding of receptor-analytes forms Host-Guest type of complex that behaves spectrally or electrochemically different from those of individual (receptor or guest) moiety [2]. Thus, several researchers are interested to develop molecular recognition/sensing systems for the specific recognition of cation or anion, or neutral organic molecules in analytical/biological/environmental samples [3]. However, sometime, it involved several steps to synthesis selective chemosensor having pseudo cavities compatible with analyte as it requires long synthetic skills [4]. Additionally, the efficiency of molecular recognition depends on many factors, of which solvent medium plays a vital role for the formation of hydrogen bonds between host molecule and guest moiety, and it develops a signal depending on the variation of the size and shape of host or guest moieties [5]. The nature of ligand attached to the binding site, and the pH of the medium are being considered essentially in the recognition process [6]. The whole issue mostly is associated with hydrophobic or hydrophilic nature of polar solvents [7].

The development of real-life materials requires robust systems, ideally, full aqueous systems would be desirable from the environmental point of view. The fluorescent response strongly depends on the energy transfer efficiency, and it is directly related to the physical distance between nanomaterials and guest ions [8–11]. As many organic chemosensors have a limited solubility in water, but it is required to be used at the same time many in aqueous matrices where relevant ions are found to be presented. Thus, it is obligated to disperse the mentioned chemosensors in aqueous systems, so it can be converted to Organic Nanoparticles (ONPs) which turned to be a stable colloidal suspension giving organic nano clusters with high surface area and great sensing potential. Thus, the Organic nanoparticles (ONPs) have been extensively investigated due to their potential use in medicine as drug delivery systems [12, 13]. To further increase selectivity and recognition properties of ONPs, the strategy is to restrict some of the coordination modes, limiting the flexibility of the free organic ligand through the bonding of the ONPs with metal or metal oxide/sulfide surfaces [14, 15]. In contrast, free ligands are highly flexible and can adopt multiple geometry depending on the steric requirement of a given metal ion. It is known that the ability of the coordination of *N*,* N*´-bis(salicylidene)ethylenediamine (salen) with different metallic ions including Cu^2+^, Ni^2+^, Zn^2+^, etc. is well established [16, 17]; however, this ability can be tuned through the immobilization agent and the composition of the supporting material. Moreover, the presence of metallic clusters such as Cu, Ag and Au in the system has further promoted the fluorescent response due to the overlapping of the *d-*orbitals with the orbital from the imine group that could support otherwise forbidden relaxation-emissive transitions [18]. For example, the decoration of a non-selective organic receptor over the surface of CdSe/ZnS (QDs) showed a unique selectivity towards cations and this ability was employed for quantification and bioimaging in biological or environmental matrices [19]. Also, solvent and pH could influence and tune recognition properties, for example, ZnO immobilized imines have been utilized for the selective recognition of Mg^2+^ [20] and substitution over aromatic rings in salen-like ligands can also modify and enhance fluorescent response towards specific ions and biomolecules [21, 22]. Thus, there is a high scientific interest on fine-tuning sensing, both to understand transfer mechanisms and for applications such as bio and chemosensors and devices based on logic gates optical memory [23].

When compared to the traditional organic fluorophores, quantum confined nanoparticles are a new class of probes that exhibit more unique optical characteristics (high quantum yield, good photostability and large Stokes shift) which have been widely exploited in the area of biosensing, immunoassays, and biological imaging [23]. Therefore, the construction of nanocomposites, composed of ONPs, and metal and metal oxides might provide additional tools to visualization by conventional microscopy in cellular imaging due to their excellent and stable fluorescence properties. It can also help in solving the transport problems for pharmacologically relevant compounds that need to be internalized. On the other hand, the thermodynamic stability of metal, metal oxides or metal chalcogenides nanoparticles can be often achieved by chelating of organic molecules on the surface of the nanoparticle [24–27]. For example, citrate or ascorbate can act as a reducing and also as capping /stabilizing agent for the metal nanoparticles. For the preparation of AuNPs, (i) citrate or ascorbate reduction [28–30], (ii) the stabilization by thiols [31–35], (iii) the reducing agents such as NaBH_4_ [31], white phosphorus [36], ethanol, and polyols are employed [37]. The advantage of trisodium citrate dehydrate or ascorbate reduces the metal ions, forming metal NPs efficiently, and it also stabilizes the size of the NPs by decreasing the large nanoparticle interface energy. However, salen is a good capping agent due to its chelating capability that can stabilize NPs efficiently; in addition, it has two imine groups, involving the bonding with metal NPs through its conjugated π electrons that support the photophysical and photochemical properties.

The ONPs of salen have been coated with metals (Ag^0^ or Au^0^), metal oxides (ZnO, CdO) or chalcogenides (ZnS, CdS) and observed the biological activity dependence with respect to the size, shape and supramolecular organization of the composites. Although there are several organic molecules based chemo-sensors for the recognition of Al^3+^ [38–50], the metal oxide nanoparticles bounded with organic molecules for the detection of Al^3+^ are limited in literature [51–54], in particular, the development of human cell images by ZnO based sensors in aqueous medium is very limited; thus, we have focused on ZnO bounded with *N*,* N´-bis*(salicylidene)ethylenediamine (salen) for the recognition of Al^3+^ in the human cells. Several studies have shown that internalization of these NPs is possible and can be, therefore, employed potentially as real time sensors in living matrices; thus, it has become a key research topic. To assess the applicability of salen ligand as a fluorescent chemosensor, in the present work, we are testing the recognition capacities and toxicological potential of metal oxides (ZnO) coated with ONPs. The most robust recognition system was further studied in its recognition capabilities under different solvents systems and applied in the real time bioimaging using U251 human cells.

## Experimental

### General Information

All analytical grade chemical reagents and solvents were purchased from Millipore-Sigma and used as received from the company without any further purification.

### Instrumentation

All UV–Vis absorption spectra are recorded on a Perkin Elmer Lambda 25 and fluorescence studies were performed on a F96 Pro. For FTIR, a Shimadzu IRTracer 100 with a QATR-10 accessory was used. The Transmission electron microscopy (TEM) images were obtained on a JEOL 2010 (200 kV) using 200 mesh C-coated copper grids. An Advance Davini 8 instrument was employed to obtain XRD patterns (CuKa = 0.1254 nm). An Olympus CKX41, USA Inverted microscope was used in cell viability and bioimaging studies; for nonlinear regression studies in cell culture GraphPad Prism 6.0e was utilized (GraphPad Software, Inc., USA).

### Synthesis of ZnO-Salen NPs

**Scheme 1:**
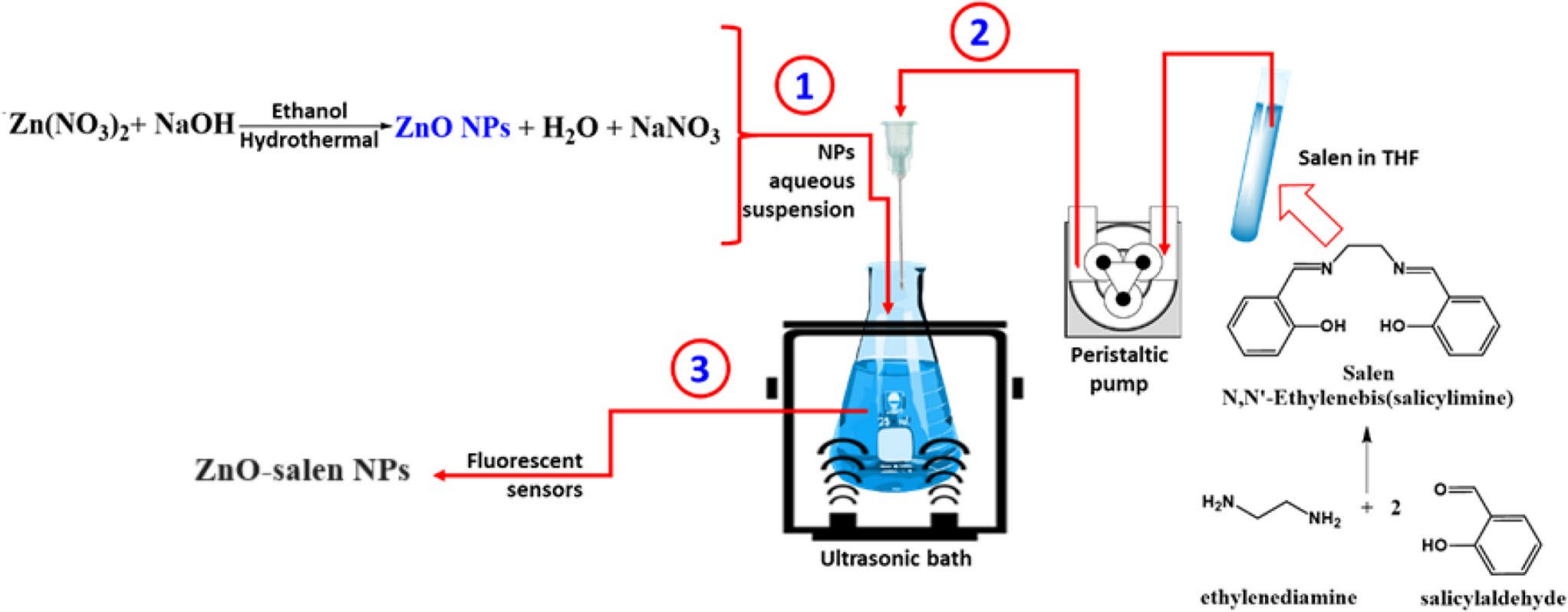
Synthetic route for ZnO-salen NPs

The experimental details of the above material preparation are illustrated in Scheme 1 and explained as follows:

#### Salen

The Salen ligand (*N*,* N´-bis(salicylidene)ethylenediamine)* was synthesized by using the procedure published elsewhere [55]. To a solution of ethylenediamine (0.60 g, 1.0 mM) dissolved in EtOH (8.0 mL), salicylaldehyde (0.244 g, 2.0 mM) dissolved in EtOH (12 mL) was slowly added, and the resulting solution mixture was stirred for 30.0 min at room temperature. The yellow crystalline solid obtained was filtrated and washed several times with diethyl ether. Yield was found to be 87%. Elemental analysis for C_16_H_16_N_2_O_2_, C, 71.62; H, 6.01; N, 10.44; O, 11.93. Found: C, 71.94; H, 5.64; N, 10.85. ^1^H NMR (300 MHz, CD_3_OD): 2CH_2_-N (4 H, d, 3.95), aromatic H (4 H, m, 6.84–6.82), aromatic H (4 H, m, 7.30–7.25), 2 N = CH (2 H, s, 8.44). ^13^C NMR (75 MHz, CD_3_OD): 166.87, 161.80, 132.28, 131.47, 118.04, 116.55, 58.59. IR: 3208 (-OH), 3047 (-CH_2_-), 1910 (-Ph), 1698 (-C = N-).

#### Salen ONPs

Organic Nanoparticles (ONPs) were prepared by a 1.0 mL of a 0.1 mM solution of Salen in THF was slowly injected (0.5 mL/min) with a peristaltic pump into 100 mL of distilled water using a 6 mm silicon tube and a 30 mm stainless steel needle under sonication (Scheme 1). The salen ONPs was characterized with TEM and X-ray diffraction pattern.

#### ZnO-Salen NPs

ZnO was prepared by a hydrothermal method (V = 70 mL,180 °C, 12 h) using a solution of Zn(NO_3_)_2_·6H_2_O (595 mg, 2.0 mmol) and NaOH (120 mg, 3.0 mmol) dissolved in ethanol. After the reaction a white product was separated out, washed with ethanol, and dried at 105 °C, 12 h. Dry ZnO (10 mg/L) was dispersed by sonication in distilled water. To this dispersion of ONPs were carried out by injecting 1.0 mL of a THF solution of Salen precursor as mentioned above. The ZnO-salen NPs was characterized with TEM and X-ray diffraction pattern.

### Metal ion Recognition Studies

The recognition of metal ions by ZnO-salen was performed by measuring the fluorescent change in the emission of the sensor system in the presence of 1.0 mM solutions containing different nitrate salts (Na^+^, Mg^2+^, Al^3+^, K^+^, Cr^3+^, Mn^2+^, Fe^3+^, Co^2+^, Ni^2+^, Cu^2+^, Zn^2+^, Sr^2+^, Ru^3+^, Ag^+^, Cd^2+^) in a full aqueous environment provided by regular distilled water (pH 7.0; HEPES 0.5 M). Additionally, the changes in the detection accuracy were studied in different MeCN-H_2_O solvent systems to give an insight into the robustness of the method towards complex media typically observed in industrial waste streams.

### Effect of pH and Temperature

The change of the fluorescent response for salen ONPs and ZnO-salen NPs was studied under different pHs, and also different temperatures in order to explore its potential application in harsh environments. ZnO-salen NPs or salen ONPs (50 mL, 0.5 mM) were suspended in water, and measured the intensity of fluorescence emission at 450 nm or 435, respectively; the effect of pH (from 3.0 to 11.0) was studied, measuring the fluorescence intensity after successive addition of HNO_3_ (0.1 M) or NH_4_OH (0.1 M); similarly, the temperature effect (20 to 80 °C, dT < 10°/min) with the intensity was also recorded.

### Sensing Recognition Studies

Whenever a system shows a positive response towards certain particular recognition for the developed ZnO-salen NPs, the stability was tested through a titration and interference test. First, the change of fluorescence intensity of the ZnO-salen NPs is measured upon adding the selected metal ion (M^n+^); the concentration (C) is progressively increased until the changes in emission (E) cease, and the system stabilizes (*dE*/*dC*0). To determine stoichiometry for ZnO-salen NPs with M^n+^ binding, a Job test was conducted where the molar fraction of the ZnO-salen NPs-M^**n+**^ system is kept constant, but the molar fraction of each component is varied, and the fluorescent response is determined. The maximum change in the fluorescence at a particular molar fraction would, therefore, correspond to the highest host**-**guest interaction.

### Toxicity by Cell Viability Studies

The human glioblastoma cell line U251 (ATCC, USA) was used to evaluate the cell toxicity of ZnO-salen NPs. Cells were grown with high-glucose DMEM medium (Biowest, USA) supplemented with 10% fetal bovine serum, 1.0 mM pyruvate and 0.1 mM non-essential amino acids (Biowest, USA) at 37 ^o^C under a 95% air and 5% CO_2_ atmosphere. 25 × 10^3^ cells were seeded in 6-well plates and treated with different concentrations (1.0 nM to 100 µM) of salen ONPs (used as vehicle), and ZnO-salen NPs, and after 24 h the cells were harvested with 1.0 mL phosphate saline buffer (PBS) with 1.0 mM EDTA and counted by a blind observer in a Neubauer chamber. Cell viability was also measured with the trypan blue dye exclusion test. The percentage of cells obtained from the ZnO-salen NPs treatment was plotted and the half maximal inhibitory concentration (IC_50_) was calculated with a non-linear regression analysis.

### Bioimaging Studies

The recognition analysis was performed by measuring the changes in fluorescent emission Human cells were cultured as previously described and 100 µL of this culture were inoculated onto glass slides to which 10 µL of the desired compound was added and a stabilization time of 30 min was established. Samples were thus observed in an Olympus FV1000 equipped with a 200–1000 nm lamp.

## Results and Discussion

### Characterization of Materials

**Fig. 1:**
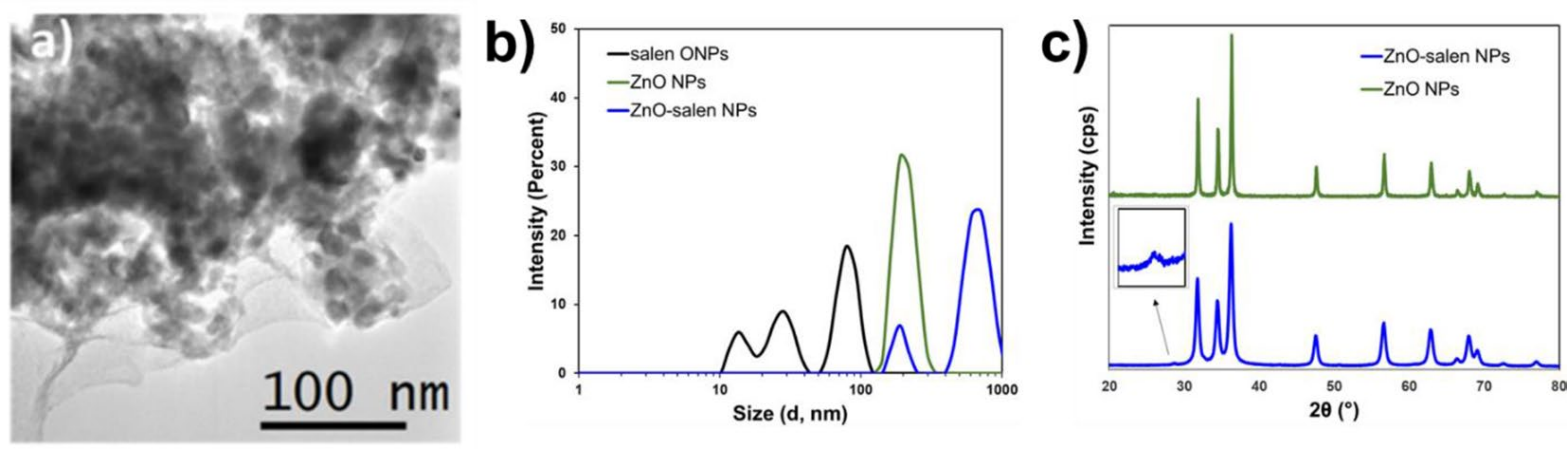
**a**) TEM of ZnO-Salen NPs; **b**) DLS determination of particle size for salen ONPs and ZnO-salen NPs; **c**) XRD pattern of bare ZnO and ZnO-salen NPs

The morphology and size of prepared sensor were analyzed by electron microscopy and Dynamic Light Scattering and observed that ZnO-salen NPs are composed of agglomeration of ZnO particles with size above 200 nm; it is noticeable that a surface coating of a low-density material was seen identifying as salen ONPs (Fig. 1a). The DLS results show that salen ONPs are composed of colloidal particles having classified in three main groups based on its size: from 15 to 40 nm with maximum population (P_max_) at 32 nm (16.3%); from 55 to 100 nm (P_max_=37 nm), accounting for 29.6% and from 65 to 160 nm (P_max_=91 nm) representing 54.4%. As bigger particles are formed, they tend to stabilize, thus being the main components of the colloidal particles. Prior to salen ONPs deposition size, zeta potential (ζ) of bare ZnO was determined. ZnO was composed of a single group of particles with size ranging from 122 to 340 nm with P_max_=220 nm (31%); dispersion stability was among the reported values being ζ =+27 mV. Upon salen ONPs formation, ZnO-salen NPs was mostly found to be agglomerated around P_max_=712 ± 200 nm, 83%. There was a minor population (13%) at P_max_= 190 nm that was identified as uncoated ZnO particles and the average ζ of this material was + 19 mV (Fig. 1b). To confirm the formation of ZnO from precursors, XRD pattern was determined after hydrothermal treatment and the main peaks for wurtzite were identified at 31.7 [100], 34.4 [002], 36.1 [101], 46.9 [102], 56.4 [110], 62.8 [103], 64.5 [200], 67.9 [112], 69.4 [201], 74.1 [004] and 77.8 [202]. After deposition of ONPs, the peaks from ZnO were conserved and observing the emergence of a new peak around 28.2° that has been previously reported in Salen-doped metal oxides (Fig. 1c).

**Fig. 2:**
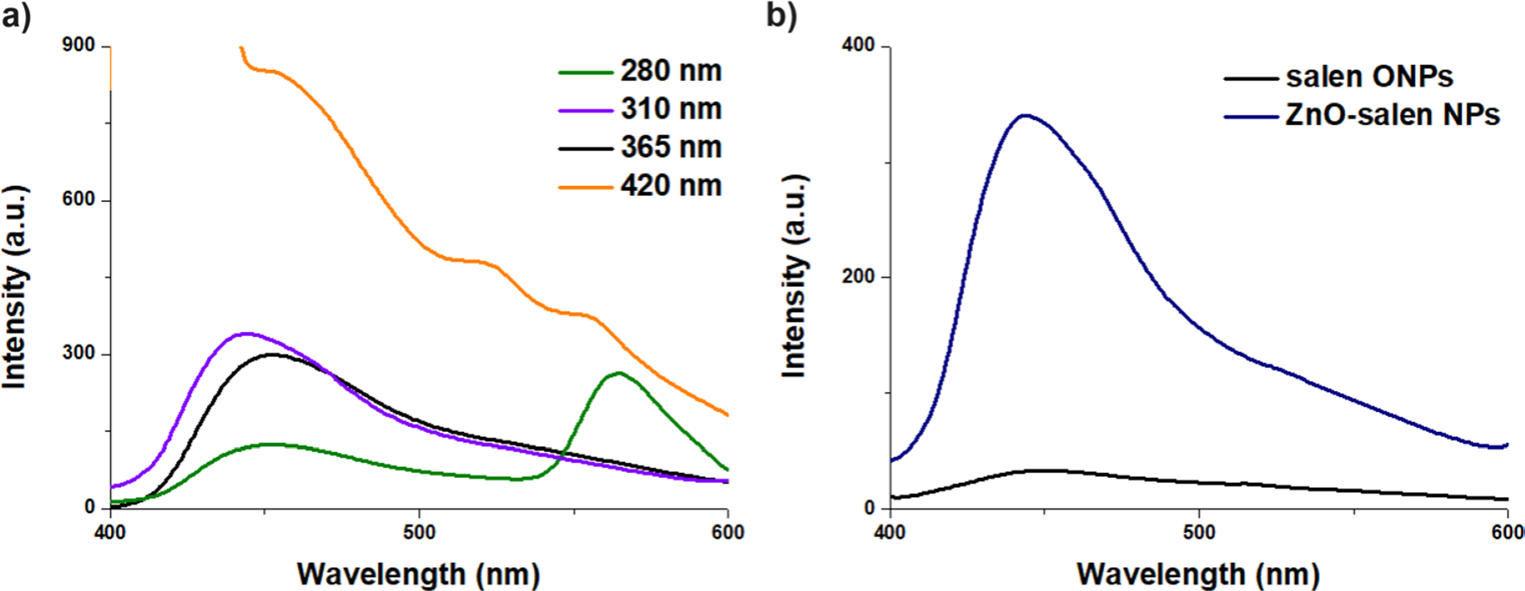
**a**) Fluorescence response of salen ONPs (1.0 mM) in aqueous media at pH 7.0 (HEPES 0.5 M) upon excitation at different wavelengths; **b**) Fluorescence spectra of salen ONPs (0.3 mM) and ZnO-salen NPs (0.3 mM) in aqueous media (HEPES 0.5 M at pH 7.0) upon excitation at 365 nm

The emission spectra of pure salen ONPs at different excitation wavelengths exhibited a maximum band at 435 nm; however, the best fluorescence intensity response has been seen for an excitation wavelength at 365 nm (Fig. 2a). On the other hand, the fluorescence emission spectrum for ZnO-salen NPs (0.5 mM) was resulted exhibiting an enhanced blue emission band at 450 nm (λ_ex_ = 365 nm), because of the coating of salen ONPs on the surface of ZnO NPs (Fig. 2b).

### Effect of pH and Temperature on the Fluorescent Response

**Fig. 3:**
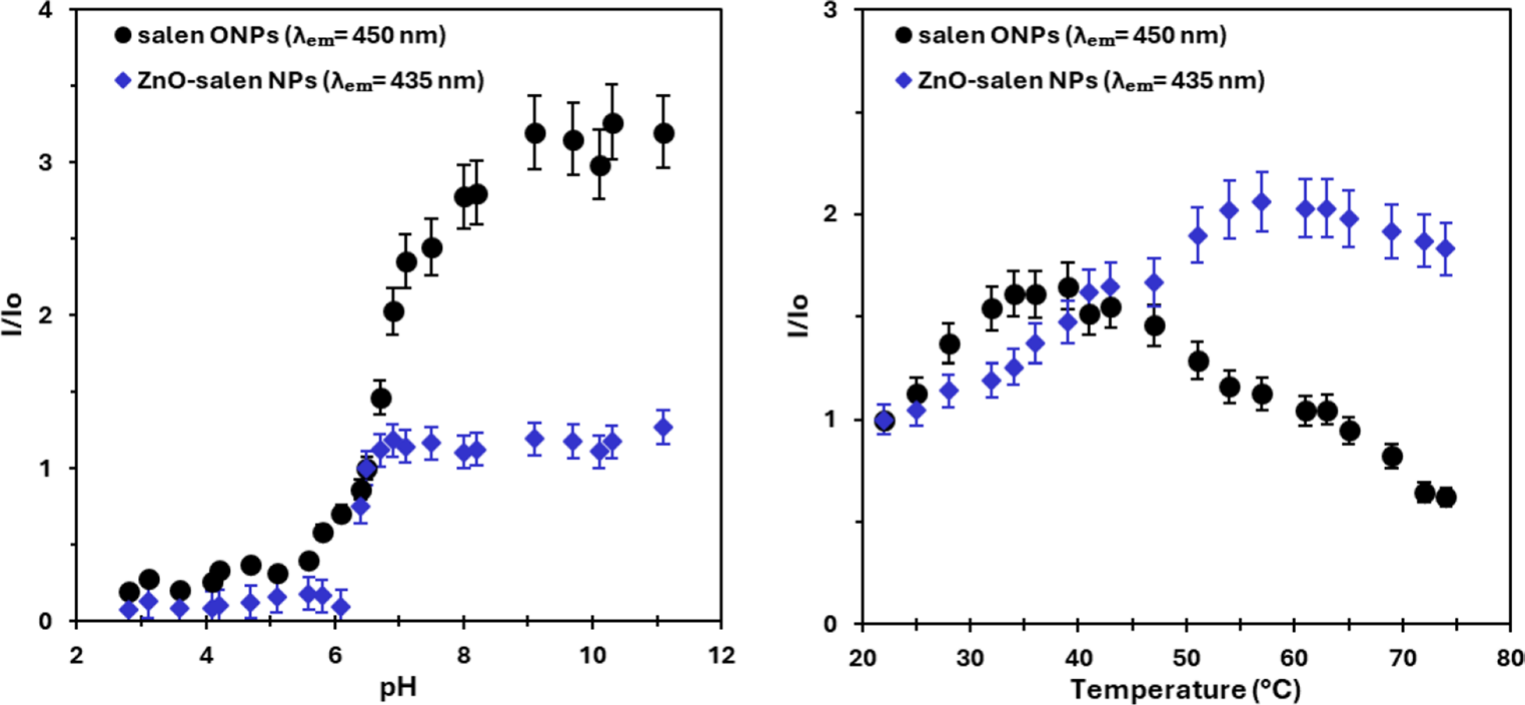
Salen ONPs and ZnO-salen NPs: Effect of pH and temperature at excitation at 365 nm

The fluorescence intensity of salen ONPs and ZnO-salen NPs was measured (I/I_0_ = 1.0 at pH 6.5 and 20 °C) at different pHs, showing the enhancement of the emission intensity in basic condition (at high pH), and the quenching of the emission in acidic medium (low pHs) (Fig. 3). For example, for salen ONPs, a maximum intensity has reached at pH 9.1 (I/I_0_ = 3.19), and for ZnO-salen NPs, it maintains a high emission intensity (an average of I/I_0_ = 1.1) from pH 7.0 to 11.0. In contrast, under acidic condition, a low fluorescence emission was observed (pH 3.6, I/I_0_ = 0.2 for salen ONPs, and I/I_0_ = 0.12 for ZnO-salen NPs) due to the protonation of imine nitrogen. This observation is consistent with the reported studies [56, 57], especially, for ZnO-based material [58] and ZnO-imine composites [20, 59].

The temperature change with the fluorescence was also monitored, and showed that after 45 °C, there was a significant quenching of fluorescence for salen ONPs in contrast to ZnO-salen NPs, where there is a considerable enhancement of fluorescence was seen (Fig. 3). For example, for salen ONPs, the temperature from 20 to 39 °C, the intensity was reached to I/I_0_ = 1.64, after which, a dramatic decrease (I/I_0_ = 0.62 at 74 °C) was noted. For ZnO-salen NPs, the emission was increased to I/I_0_ = 2.06 at 57 °C, and then, it was gradually decreased to I/I_0_ = 1.82 at 74 °C. This is probably due to the evaporation of solvent from the mesoporous structure of ZnO at high temperature, and it allows its active sites for the interaction of salen ligand. However, at high temperature, it is highly possible that imine groups can undergo a hydrolysis, affecting the fluorogenic activity.

### Cationic Recognition Tests for Sequential Selective Sensing of Al^3+^ and K^+^ by ZnO-Salen NPs

**Fig. 4:**
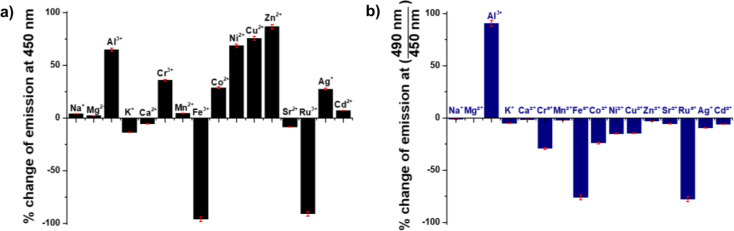
Metal binding tests (1.0 mM): (**a**) salen ONPs (0.1 mM) and (**b**) ZnO-salen NPs (0.1 mM) in water buffered at pH 7.0 (λ_ex_ = 365 nm)

**Fig. 5:**
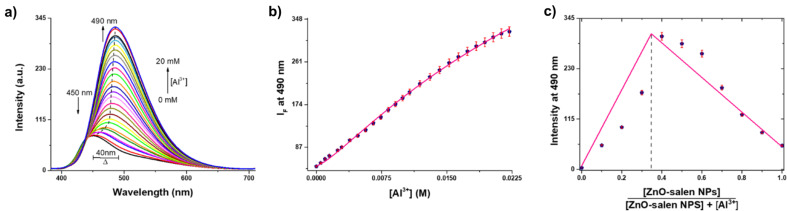
**a**) Spectrofluorimetric titration of buffered aqueous solution (0.5 M, HEPES at pH 7.0) of ZnO-salen NPs (0.1 mM) by Al^3+^. The arrows show the direction of the spectral changes. **b**) Profile of emission at 490 nm for increasing amounts of Al^3+^ (average of triplicate experiments). The line was obtained by fitting the data to a 2:1 (Al^3+^:ZnO-salen) binding model using Eq. (1). **c**) Stoichiometric analysis of ZnO-salen NPs by Job’s plot with Al^3+^ at pH = 7.0

In the cationic recognition study, ZnO-salen was employed as chemosensor for the recognition of Al^3+^, and observed a significant florescence change only for Al^3+^ after testing several metal ions (Na^+^, K^+^, Mg^2+^, Sr^2+^, Al^3+^, Cr^3+^, Mn^2+^, Fe^3+^, Ru^3+^, Co^2+^, Ni^2+^, Cu^2+^, Ag^+^, Zn^2+^, and Cd^2+^) (Fig. 4b). For the samples, the solution was excited at 365 nm and the fluorescence emission was observed in the visible region. This same test was also performed for salen ONP; however, no selectivity was observed after using the same cations in the form of nitrate salts (Fig. 4a). Additionally, an identical test was performed for anions using ZnO-salen NPs as chemosensor, but no such changes were found after using the following anions in the form of tetrabutylammonium salts (F^-^, I^-^, Cl^-^, Br^-^, HSO_4_^-^, ClO_4_^-^, AcO^-^ and H_2_PO_4_^-^). Thus, this ZnO-salen system is suitable only for Al^3+^ (Fig. 4b), consisting with the titration of Al^3+^ vs. ZnO-salen NPs (Fig. 5), where for the successive addition of Al^3+^ to ZnO-salen NPs (0.1 mM), an enhancement of fluorescence intensity at 490 nm was observed. This means that the binding nature of ZnO-salen (receptor) with Al^3+^ enhances the fluorescence intensity. This can be explained as follows:

(i) The addition of Al^3+^ to the solution of ZnO-salen undergoes a red-shift in the peak, probably due to strong coordination of Al^3+^ ion with ZnO-salen during the high concentration of Al^3+^ in the solution. The PET tuned to ‘ON’ from the OH if any metal ion binds to receptor by using the OH binding site, and it increases the fluorescence intensity on the same wavelength. Nevertheless, if any metal ion also binds to CH = N, a close binding to fluorophore leads to the shift the wavelength position because of changing in the modulation of transfer charge transfer.
(ii) The excitation of ZnO-salen without the Al^3+^ presence, the energy level of highest occupied molecular orbital (HOMO) of the receptor (before binding with ZnO-salen) would be higher compared to that of the excited fluorophore where HOMO would have half-filled. So, this electronic energy configuration encourages a rapid electron transfer from the receptor to the excited-state fluorophore, and it favors a quenching of fluorescence intensity. Whereas, if the interaction of ZnO-salen (ionophore) with Al^3+^, the receptor’s HOMO energy level is turned to be lower, inhibiting the receptor electron transfer to the HOMO of excited-state fluorophore, and it causes the fluorescence mode to be “Turn-On”.

After establishing Al^3+^ recognition capacity by ZnO-salen NPs, Job’s plot measurements based on the emission were performed. The emission has exhibited at 490 nm with a maximum when the mole fraction was 0.4 (Fig. 5c), which belongs to a 1:2 binding stoichiometry for the system of ZnO-salen NPs with Al^3+^. The Fig. 5a illustrates the family of emission spectra obtained when an aqueous solution (HEPES, 0.5 M at pH = 7.0) of ZnO-salen NPs (0.1 mM) is titrated with Al^3+^ ions (0–20 mM) and the Fig. 5b shows the ratiometric response of intensity at 490 nm/450 nm on progressive addition of Al^3+^. The profile curve of the ratiometric change of emission at 490 nm/450 nm remained unchanged when the concentration of Al^3+^ was 20 mM. The titration profile showed a sigmoidal curve indicating the formation of two-step complexation process. This profile could be well fitted to a 2:1 (ZnO-salen: Al^3+^) binding model using a nonlinear least-squares treatment with Eq. (1) [60, 61] to give a global formation constant of *β*_*2*_ = *K*_*1*_*·K*_*2*_ = 6.61 × 10^3^ ± 0.04, where *K*_*1*_ and *K*_*2*_ are the complexation constants for the first and second Al^3+^ atom, *I*_*F*_ is the observed intensity at 490 nm and *I*_*1*_ and *I*_*2*_ are the emission intensity of the 1:1 and 1:2 stoichiometry, respectively. The Job’s plot confirmed this stoichiometry (Fig. 5c).

1 $${\textbf{\textit{I}}}_{\textbf{\textit{F}}}=\frac{\left({\textbf{\textit{I}}}_{\textbf{0}}+{\textbf{\textit{I}}}_{\textbf{1}}{\cdot}{\textbf{\textit{K}}}_{\textbf{1}}{\cdot}\left[{\textbf{\textit{A}}}{\textbf{\textit{l}}}^{{\textbf{3+}}}\right]+{\textbf{\textit{I}}}_{\textbf{2}}{\cdot}{\textbf{\textit{K}}}_{\textbf{1}}{\cdot}{\textbf{\textit{K}}}_{\textbf{2}}{\cdot}\left[{\textbf{\textit{A}}}{\textbf{\textit{l}}}^{{\textbf{3+}}}\right]^{\textbf{2}}\right)}{1+{\textbf{\textit{K}}}_{\textbf{1}}{\cdot}\left[{\textbf{\textit{A}}}{\textbf{\textit{l}}}^{{\textbf{3+}}}\right]+{\textbf{\textit{K}}}_{\textbf{1}}{\cdot}{\textbf{\textit{K}}}_{\textbf{2}}{\cdot}\left[{\textbf{\textit{A}}}{\textbf{\textit{l}}}^{{\textbf{3+}}}\right]^{\textbf{2}}}$$

**Fig. 6:**
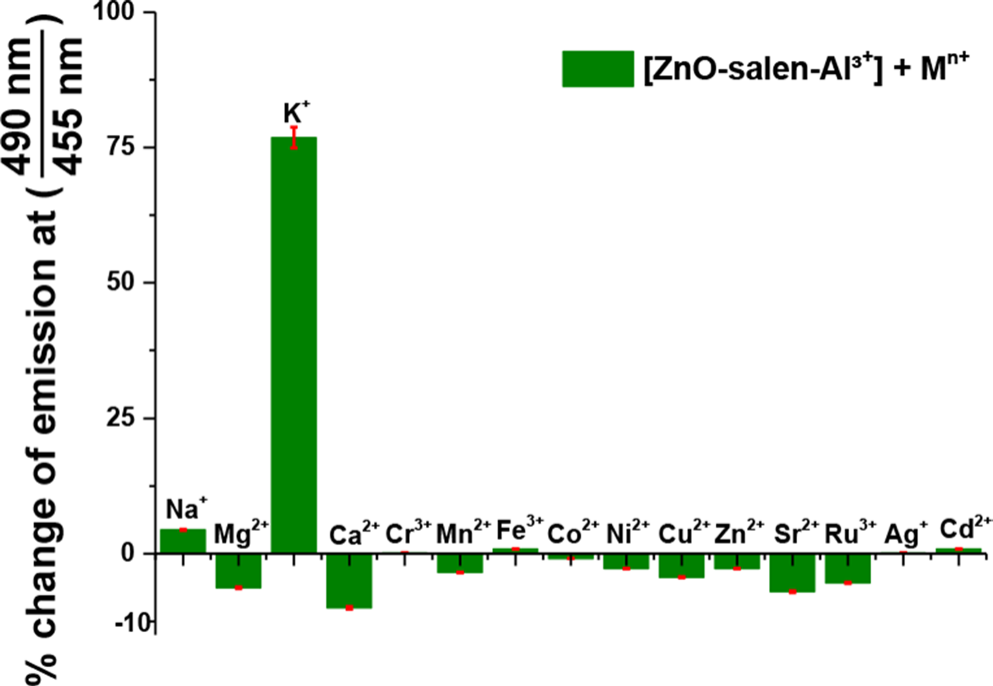
Metal binding tests (1.0 mM) of ZnO-salen-Al^3+^ (0.1 mM ZnO-salen + 0.2 mM Al^3+^) in water buffered at pH 7.0 (λ_ex_ = 365 nm)

Notably, there is a linear dependence in the fluorescence intensity for ZnO-salen NPs with Al^3+^ at different concentrations (range of 0–6.0 mM, R^2^ = 0.99832) in an aqueous solution, and it gives a limit of detection (LOD) of 36.51 µM. The LOD was calculated using the formulae LOD = 3σ/s, where σ = standard deviation of blank luminescence intensity and s = slope of the calibration plot, *s* = 13.15 (± 0.18) × 10^3^ M (Figure S4). For practical applications, the chemosensors must have not only good optical response and affinity, but also selectivity in the presence of coexistent potential interferences in real samples. Therefore, a selectivity experiment of ZnO-salen-Al^3+^ (0.1 mM ZnO-salen + 0.2 mM Al^3+^) towards the possible interfering metal cations was carried out at pH = 7.0. The addition of these cations to aqueous suspensions of ZnO-salen NPs has produced a negligible change in its emission, except for the addition of K^+^, for which, an enhancement of fluorescence intensity was observed (Fig. 6).

**Fig. 7:**
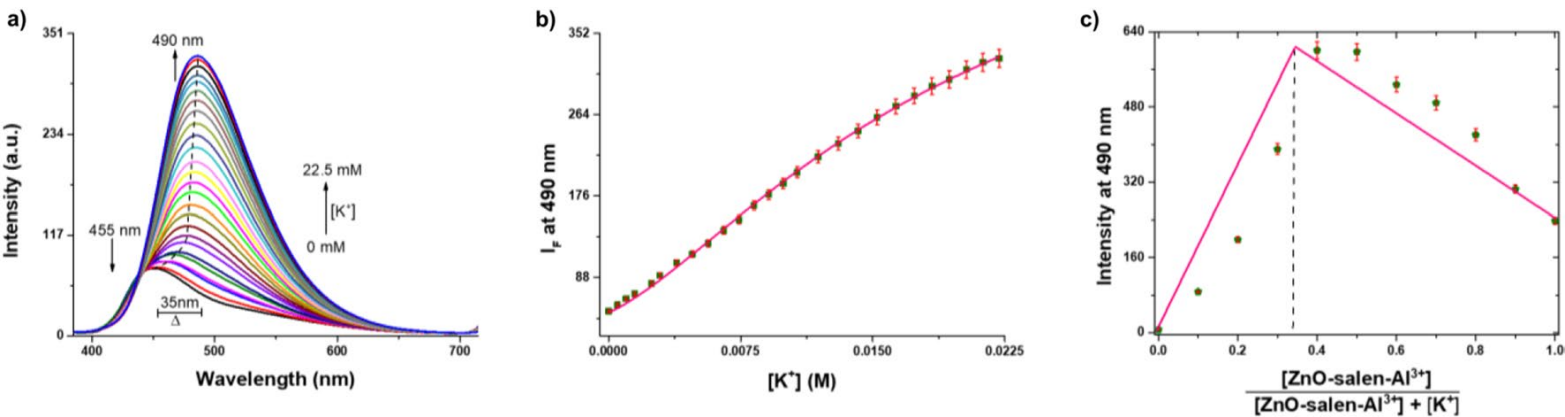
**a**) Spectrofluorimetric titration of buffered aqueous solution (0.5 M, HEPES at pH 7.0) of ZnO-salen-Al^3+^ (0.1 mM) by K^+^. The arrows show the direction of the spectral changes. **b**) Profile of emission at 490 nm for increasing amounts of K^+^ (average of triplicate experiments). The line was obtained by fitting the data to a 2:1 (K^+^:ZnO-salen-Al^3+^) binding model using Eq. (1). **c**) Stoichiometric analysis of ZnO-salen-Al^3+^ by Job’s plot with K^+^ at pH = 7.0

To learn more about the properties of ZnO-salen-Al^3+^ as a receptor for K^+^, fluorescence titration was carried out (Fig. 7a). The addition of increasing amounts of potassium to the suspension of ZnO-salen-Al^3+^ has further increased the emission intensity at 490 nm, which was attributed to the formation of an ZnO-salen-Al^3+^-K^+^ complex with a *β*_*2*_ = 3.71 × 10^3^ (Fig. 7b, Eq. 1) and a host guest ratio of 1:2 determined from Jobs plot in aqueous environment (HEPES, 0.5 M at pH = 7.0) (Fig. 7c). There is a linear dependence in the fluorescence intensity for ZnO-salen-Al^3+^ with K^+^ at different concentrations (range of 0–7.0 mM, R^2^ = 0.9987) in an aqueous solution, and it gives a limit of detection (LOD) of 17.39 µM (Figure S5). The observed fluorescent recognition results could be explained as follow: The stepwise addition of K^+^ to ZnO-salen-Al^3+^ leads to the formation of intramolecular coordination sphere using both O-H and C = N linkages, thus PET is cancelled from O-H and Charge Transfer band is also affected.

Additionally, the relative quantum yields of ZnO-salen NPs with Al^3+^ and K^+^, in HEPES buffer at pH 7.0 were calculated using quinine sulfate in 0.1 M H_2_SO_4_ as the reference (Φ_ref_ = 0.540) according to the Eq. (2), where the subscripts “x” and “ref” denote sample and reference standard. *F* is the spectrally integrated photon flux at the detector, i.e., the area under the emission spectrum, and *A* is absorption factor provides the fraction of the excitation light absorbed by the sample and reference standard. The Eq. 2 contains also a refractive index correction term (η) that has to be applied if different solvents are used for sample and standard. The relative quantum yields of ZnO-salen, ZnO-salen-Al^3+^ and ZnO-salen-Al^3+^-K^+^ are Φ = 0.098, Φ = 0.109 and Φ = 0.120, respectively.

2 $${\phi}_{\textbf{\textit{x}}}=\phi_{{\textbf{\textit{ref}}}}\left(\frac{{\textbf{\textit{A}}}_{{\textbf{\textit{ref}}}}}{{\textbf{\textit{A}}}_{\textbf{\textit{x}}}}\right)\left(\frac{{\textbf{\textit{F}}}_{\textbf{\textit{x}}}}{{\textbf{\textit{F}}}_{{\textbf{\textit{ref}}}}}\right)\left(\frac{\eta_{\textbf{\textit{x}}}}{\eta_{{\textbf{\textit{ref}}}}}\right)^{\textbf{2}}$$

**Scheme 2:**
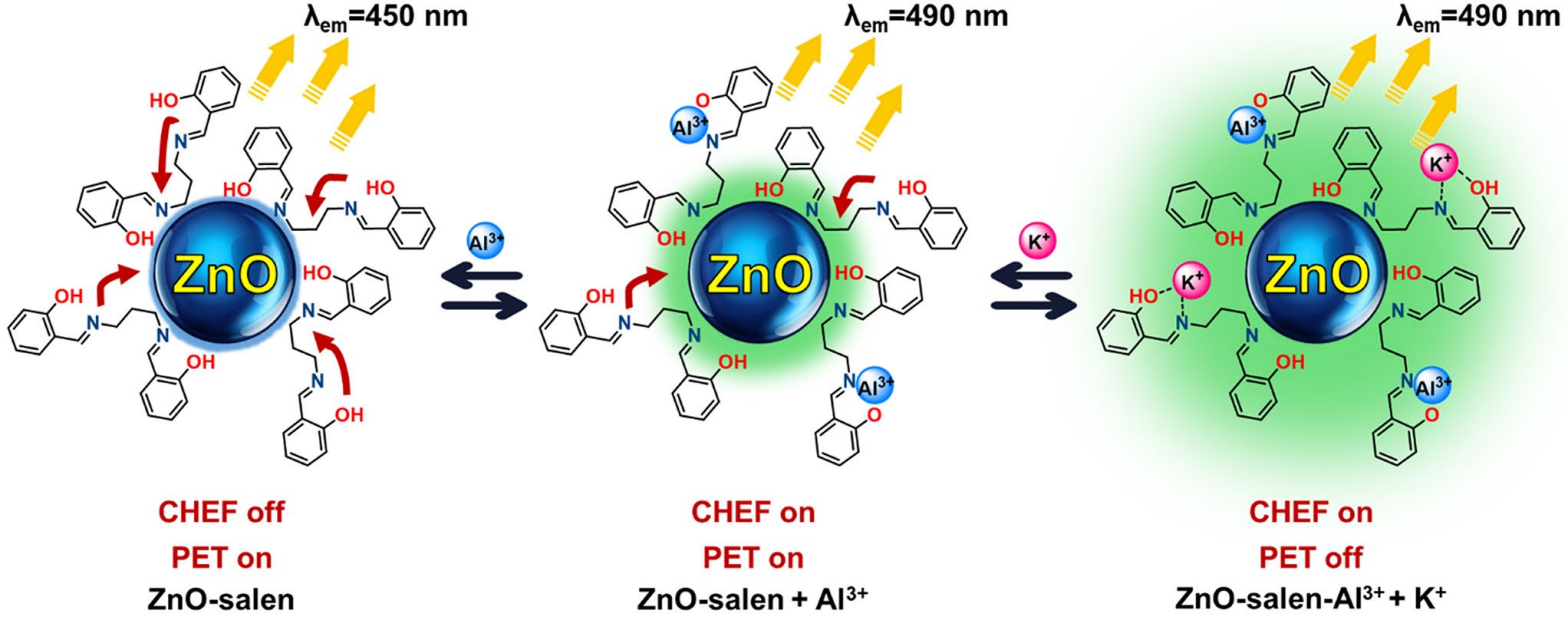
Proposed sequential sensing mechanisms of Al^3+^ and K^+^ by ZnO-salen NPs

The relative quantum yields show that there is an enhancement of the emission intensity after the sequential addition of Al^3+^ and K^+^. This photophysical phenomenon is attributed to chelation-enhanced fluorescence (CHEF) mechanism operating between Al^3+^ and ZnO-salen; thus, it, subsequently, cancels the photoinduced electron transfer (PET) process, promoted by ion-dipole- electrostatic interaction that presented between potassium ions and salen ligand (imine and phenol groups) [62]. According to fluorimetric titrations, it follows a turn-on fluorescent detection mechanism as it consists with job plots and the fluorescence quantum yields (Scheme 2).

**Scheme 3:**
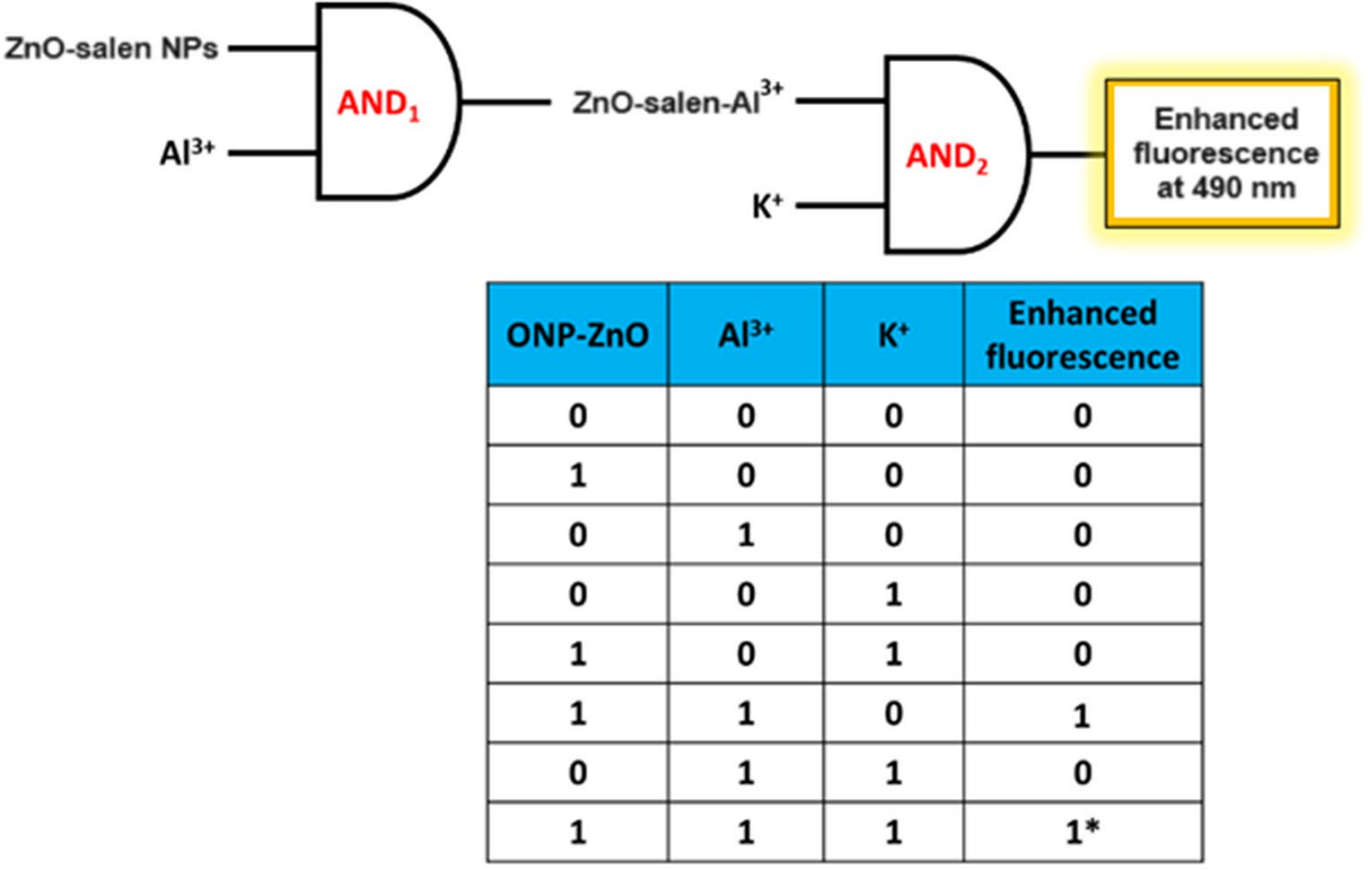
Two successive AND logic gates as sensing mechanisms for sequential detection of Al^3+^ and K^+^ by ZnO-salen NPs

Logic gates for CDs are important for detecting various analytes and generally most of the logic gates are fundamentally based on fluorescence spectroscopy because of their sensitiveness. According to the results, it was found that a logic gate system exists in the recognition of K^+^ by ZnO-salen in presence of Al^3+^ ions. This means that the enhancement of fluorescence intensity for K^+^ with ZnO-salen NPs was observed only if the Al^3+^ ions are presented; however, the intensity was low if other combinations of ZnO-salen NPs with K^+^ are used. First, an AND logic gate was performed through the binding of ZnO-salen NPs with Al^3+^ ions [A = 1, B = 1], turning on the fluorescence was seen at 490 nm. In another AND logic gate [A = 1, B = 1, C = 1], the fluorescence intensity of ZnO-salen-Al^3+^ increased when K^+^ ions are added. This indicates that ZnO-salen NPs first recognize Al^3+^ ions and then detect K^+^ ions (Scheme 3).

### Toxicity Studies of ONPs in Human U251 Cells

**Fig. 8:**
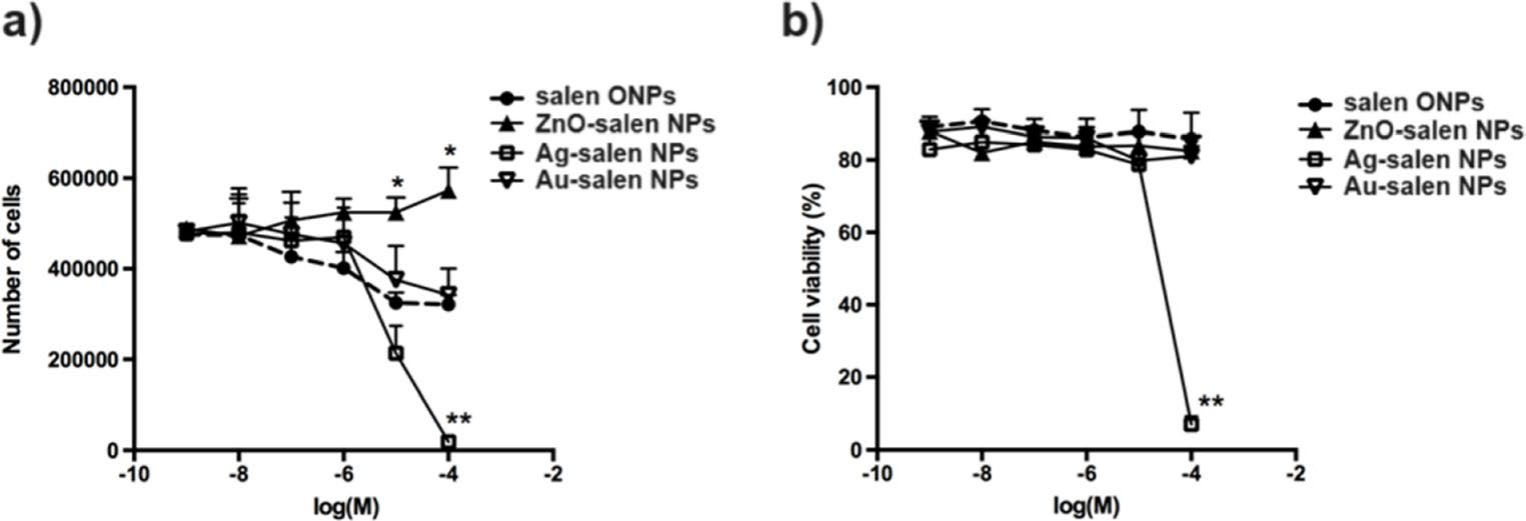
Effects of salen ONPs based materials concentration (1.0 nM to 100 µM) on U251 cell number and viability upon 24 h treatment: **a**) number of cells and **b**) viable cells after treatment with the different concentrations (log M). *P < 0.05 vs. salen ONP and Ag-salen NPs, **P < 0.01 vs. all other groups, mean ± S.E.M., *n* = 4

The human glioblastoma derived cell line U251 was used for the cytotoxic evaluation of different salen ONPs coupled to various metals (ZnO, Au^0^, and Ag^0^). For this purpose, different concentrations of the salen ONPs were used (1 nM to 100 µM) and the number of cells as well as their viability was evaluated. Salen ONPs and Au-salen NPs slightly decreased the number of cells in a concentration-dependent manner, while Ag-salen NPs significantly reduced it at 100 µM. Interestingly, ZnO-salen NPs did not reduce the number of cells, and on the contrary, it significantly increased it as compared with the salen ONP treated cells from 10 µM to 100 µM (Fig. 8a). Despite the effects on the number of cells, the cell viability was maintained (85–90%) for salen ONP, ZnO-salen NPs, and Au-salen NPs treatments. However, Ag-salen NPs markedly reduced cell viability to 3% when the highest concentration was used (100 µM) (Fig. 8b). These data suggest that high concentrations of ZnO-salen NPs increase the proliferation capabilities of the cells without altering their viability, while Ag-salen NPs promotes cell death through a cytotoxic effect. Once the cytotoxic effect of Ag-salen NP was evaluated, the data were used to calculate the half maximal inhibitory concentration, IC_50_ = 20 µM according to the dotted line representing the non-linear regression model shown in Figure S6. It is suggested that the acidic environment of the lysosomes promotes the degradation of the ONP coating with the subsequent release of the metal and metal oxide particles. In case of Ag and Au clusters, they oxidize into toxic metal ions, although their release depends on the coating structure [63, 64]. Once inside the cell, metal ions can disrupt the cell membranes, generate reactive oxygen species (ROS), damage protein’s structure and function, and disrupt the DNA structure, leading eventually to cell death [65].

Our results show that 100 µM Ag-salen NPs reduces the number and the viability of U251 cells. Elemental silver (Ag^0^) can be oxidized into Ag^+^ under physiological conditions which is known to have toxic effects in the human colorectal cancer cells Caco-2 [66] and reduce cell viability and promote cell death in the human myeloid leukemia cell line SHI-1 [67]. In the glioblastoma cell line U87 coated Ag nanoparticles induce DNA damage, increase oxidative stress, mitochondrial dysfunction, and cell death [68]. The reduced cell viability observed for the U251 cells should be through Ag^+^ accumulation inside the cell and DNA fragmentation that eventually leads to cell death. In case of salen ONPs and Au-salem NPs, there is a tendency to reduce the number of cells in a concentration-dependent manner without affecting the cell viability. This suggests a possible cell cycle arrest and cell toxicity could emerge only under very high concentrations. Interestingly, treatment with 10 and 100 µM of ZnO-salen NPs increased the number of cells. ZnO nanoparticles are known to induce ROS production in the cells [69], and reports show that in mouse macrophages ZnO nanoparticles uptake elicit a cytotoxic response that does not depend on the cellular increase in ROS [70]. In contrast, in human myoblastoma cancer cells, ZnO nanoparticles increase ROS production and caspase-3 activity thus promoting cell death [71]. However, a low level of ROS generation promotes the proliferation of several types of cancer cells and, remarkably, the overexpression of mitochondrial protein Romo1 in glioblastoma cells is associated with ROS production and cell proliferation [72, 73]. Therefore, ZnO-salen NPs internalization could be promoting an increase in ROS production that should be favoring U251 cell proliferation.

### Application of ONP-ZnO in Bioimaging

**Fig. 9:**
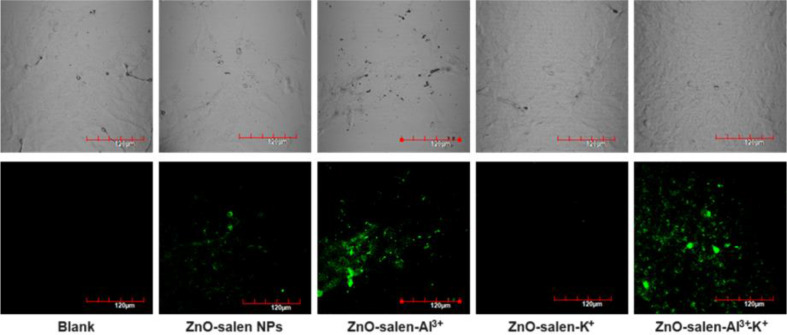
Application of ZnO-salen NPs as chemosensor for recognition of Al^3+^ and K^+^ by bioimaging in human glioblastoma derived cell line U251

From Fig. 9, it can be observed that fluorescent emission within cells upon excitation at ~ 490 nm follows the trends previously observed in aqueous solution, which indicated that the interaction between ZnO-salen NPs and analytes is strong enough to preserve its activity in biological media, where multiple anions and cations are usually present. ZnO-salen NPs that are present in the cell culture are internalized through endocytosis and stored in lysosomal compartments. In presence of ZnO-salen NPs, fluorescence displayed is mainly centered in the nucleus and its boundaries while in presence of Al^3+^, fluorescence is extended to plasmatic region. These changes can be explained since the cytosol is a region where Al^3+^ ions can be freely dispersed and thus interaction with ZnO-salen NPs could take place before this system is attached to nucleus borders. For addition of ZnO-salen NPs and K^+^, no fluorescence was observed at all, but in case of sequential addition of Al^3+^ and K^+^, a slight enhancement in fluorescence intensity is observed; also, the emission is observed uniformly distributed among cytosol which confirms that dispersed ions in cytosol contribute to interact with ZnO-salen NPs. It is important to observe that changes in fluorescence between ZnO-salen-Al^3+^ and ZnO-salen-Al^3+^-K^+^ are not as intense as observed in vitro; this is understandable since most living cells contain K^+^ and thus an instant interaction and interference would be expected towards freshly formed ZnO-salen-Al^3+^ [74, 75].

## Conclusions

Salen Organic Nanoparticles and ZnO-salen hybrid were prepared and studied their photophysical and photochemical properties. The results reveal that ZnO-salen selectively recognizes Al^3+^ ions in aqueous medium by enhancing the fluorescence intensity. The effect of pH and temperature is also performed, showing an enhancement of fluorescence intensity at high pH, yielding a maximum emission at pH 9.1 for salen ONPs, and pH 9.7 for ZnO-Salen; at low pH, a quenching of the intensity is observed, due to the protonation of the ligand. With the increase of temperature, the increase of fluorescent intensity is noticed, namely, it has reached a maximum fluorescence at 39 °C for Salen ONPs, while for ZnO-salen, it was at 57 °C. With the presence of K^+^ in the medium, it is further intensified the emission for the [ZnO-salen-Al^3+^] system. The observed binding constants are *β*_*2*_(Al^3+^) = 6.61 × 10^3^, and *β*_*2*_(K^+^) = 3.71 × 10^3^ (subsequent recognition of K^+^ by ZnO-salen), and the limit of detection found to be 36.51 µM for Al^3+^ and 17.39 µM for K^+^. The above system is employed to recognize Al^3+^ and K^+^ in cells by developing the cell images for glioblastoma U251, and observed the brightening of the image for [ZnO-salen-Al^3+^] + K^+^ ions. Cell toxicity analysis shows the cell viability of 85% for the ZnO-salen.

## Electronic Supplementary Material

Below is the link to the electronic supplementary material.

Supplementary Material 1

## Data Availability

No datasets were generated or analysed during the current study.
