# Supplementary material for: ZnO-Salen NPs Employed as Chemosensor for Detection of Al3+ and K+ in Aqueous Medium, Developing Human Cell Images
Source: J Fluoresc. 2024 Aug 31;35(7):5319–31. doi: 10.1007/s10895-024-03913-4 (PMC12325485; doi:10.1007/s10895-024-03913-4)
Supplement: Supplementary file 1 — Supplementary Material 1 [file 10895_2024_3913_MOESM1_ESM.docx]

**ZnO-Salen NPs employed as chemosensor for detection of Al^3+^ and K^+^ in aqueous medium, developing human cell images**

Carlos Alberto Huerta-Aguilar,^a^ Iván J. Bazany-Rodríguez,^b^ Valeria Hansberg-Pastor,^a^ Ignacio Camacho-Arroyo,^c^ Iván Alejandro Reyes-Dominguez,^d^ Pabel Antonio Cervantes-Avilés,^a^ and Pandiyan Thangarasu *^b^

1. School of Engineering and Sciences, Tecnológico de Monterrey, 72456, Puebla, Mexico.
2. Faculty of Chemistry, Universidad Nacional Autónoma de Mexico, 04510, CDMX, Mexico.
3. Unidad de Investigación en Reproducción Humana, Instituto Nacional de Perinatología-Facultad de Química, Universidad Nacional Autónoma de México
4. Instituto de Metalurgia, Universidad Autónoma de San Luis Potosí, 78210, San Luis Potosí, Mexico.

**Supplementary Material**

**
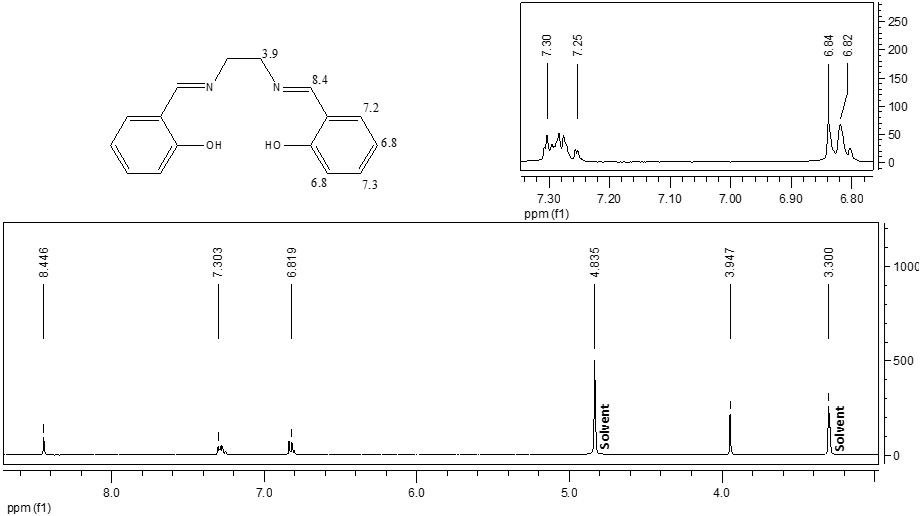
**

**Figure S1.** ^1^H RMN spectra (300 MHz, solvent: methanol-*d_4_*) of salen ligand.

**
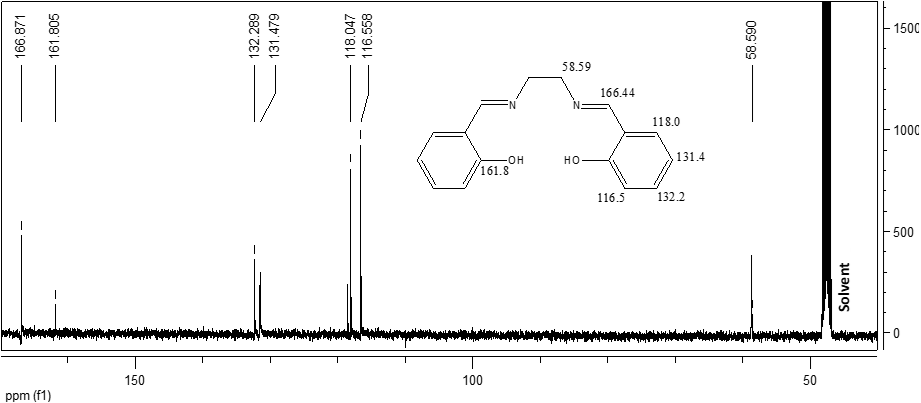
**

**Figure S2.** ^13^C RMN spectra (75 MHz, solvent: methanol-*d_4_*) of salen ligand.

**
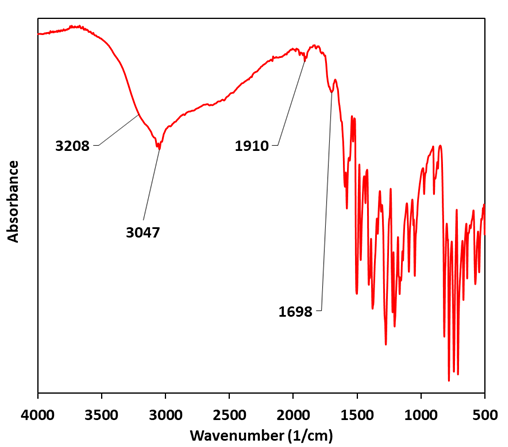
**

**Figure S3.** FT IR characterization of salen ligand.

**Figure S4.** Linear dependence in the fluorescence intensity for ZnO-salen NPs with Al^3+^ at different concentrations (range of 0–6.0 mM, R^2^ = 0.99832) in an aqueous solution pH 7.0.

**Figure S5.** Linear dependence in the fluorescence intensity for ZnO-salen-Al^3+^ with K^+^ at different concentrations (range of 0–7.0 mM, R^2^ = 0.9987) in an aqueous solution at pH 7.0.


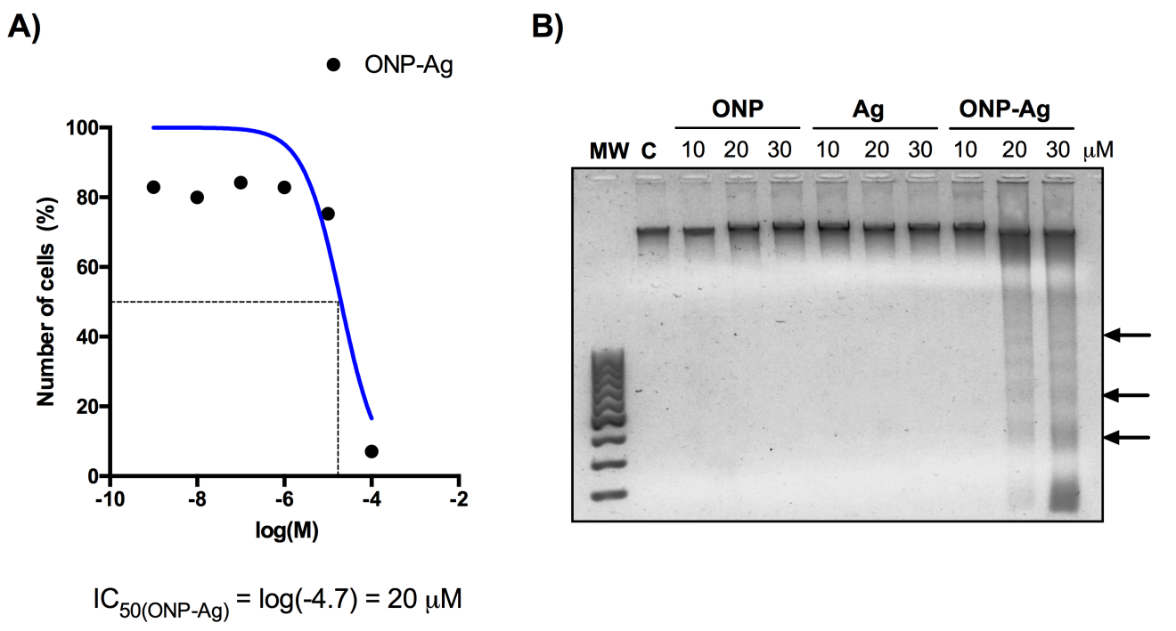


**Figure S6.** The percentage of living U251 cells treated with different concentrations of ONP-Ag (log M) utilized to calculate the IC_50_ using a non-linear regression analysis.
